# Use of Biomonitoring Data to Evaluate Methyl Eugenol Exposure

**DOI:** 10.1289/ehp.9057

**Published:** 2006-06-12

**Authors:** Steven H. Robison, Dana B. Barr

**Affiliations:** 1 The Procter & Gamble Company, Central Product Safety Division, Product Safety and Regulatory Affairs, Miami Valley Laboratories, Cincinnati, Ohio, USA; 2 Centers for Disease Control and Prevention, National Center for Environmental Health, Division of Laboratory Sciences, Organic Analytical Toxicology, Atlanta, Georgia, USA

**Keywords:** biomonitoring, exposure assessment, methyl eugenol, risk assessment

## Abstract

Methyl eugenol is a naturally occurring material found in a variety of food sources, including spices, oils, and nutritionally important foods such as bananas and oranges. Given its natural occurrence, a broad cross-section of the population is likely exposed. The availability of biomonitoring and toxicology data offers an opportunity to examine how biomonitoring data can be integrated into risk assessment. Methyl eugenol has been used as a biomarker of exposure. An analytical method to detect methyl eugenol in human blood samples is well characterized but not readily available. Human studies indicate that methyl eugenol is short-lived in the body, and despite the high potential for exposure through the diet and environment, human blood levels are relatively low. The toxicology studies in animals demonstrate that relatively high-bolus doses administered orally result in hepatic neoplasms. However, an understanding is lacking regarding how this effect relates to the exposures that result when food containing methyl eugenol is consumed. Overall, the level of methyl eugenol detected in biomonitoring studies indicates that human exposure is several orders of magnitude lower than the lowest dose used in the bioassay. Furthermore, there are no known health effects in humans that result from typical dietary exposure to methyl eugenol.

Methyl eugenol [1,2-dimethoxy-4-(2-propenyl)benzene (CAS no. 93-15-2), structure shown in Figure 1] is a member of a family of chemicals known as allyl alkoxy-benzenes, which include other naturally occurring materials such as isoeugenol, eugenol, estragole, and safrole. All these compounds typically enter the diet via a variety of different food sources, including spices (nutmeg, allspice), herbs (basil, tarragon), bananas (Jordan et al. 2001), and oranges (MacGregor et al. 1974). Many of these compounds are also found as components of natural oils used in perfumes (Smith et al. 2002). In addition there are other potential sources of exposure to methyl eugenol, including agriculture (Vargas et al. 2000), consumption of wine (De Simon et al. 2003), and as part of the ambient background in air and water (Barr et al. 2000).

Given the broad potential for exposure resulting from both dietary and consumer product use and its structural similarity to other carcinogenic allyl alkoxybenzenes, methyl eugenol was nominated for study by the National Toxicology Program (NTP) at the National Institute of Environmental Health Sciences. The NTP evaluated methyl eugenol in a rodent bioassay using oral gavage as the route of administration (NTP 2000). Based on the results of the bioassay, the NTP concluded that there was clear evidence of car-cinogenicity in F344 rats and B6C3F_1_ mice. This conclusion was based on increases in male and female rats of hepatocellular carcinoma and hepatocholangiocarcinoma, neuroendocrine tumors of the glandular stomach, and the observation of other tumor types in several other tissues, and the primary tumors observed in B6C3F_1_ mice were hepatocellular carcinoma in male and female mice and increased neuroendocrine tumors of the glandular stomach in male mice. Although toxicologic end points have been established in animals, searches of the literature have identified no clinical studies or epidemiology data to provide perspective on whether there are health effects associated with long-term consumption of methyl eugenol by humans.

To provide human exposure data in support of NTP’s assessment of methyl eugenol, the Centers for Disease Control and Prevention (CDC) measured methyl eugenol in a non-representative subset of adult serum samples collected as a part of the Third National Health and Nutrition Examination Survey (NHANES III, 1988–1994) (CDC 2004). The mean serum methyl eugenol concentration in this subset was approximately 24 pg/g serum (whole weight), with concentrations ranging from < 3.1 to 390 pg/g serum (whole weight) (Barr et al. 2000). The human elimination kinetics were also evaluated. Volunteers ingested gingersnap cookies containing a total of 216 μg methyl eugenol (Schecter et al. 2004). Samples taken after an overnight fast and before the gingersnap meal had measurable levels of methyl eugenol. About 15 min after ingesting the gingersnaps, the mean concentration of methyl eugenol peaked at 54 pg/g serum (whole weight), then fell to a mean level of about 25 pg/g serum (whole weight) after 2 hr (Schecter et al. 2004). The results of this study suggest that low levels of methyl eugenol are present in the blood after an oral dose and that the levels rapidly decline. Because measurements were not made on any elimination matrices (e.g., urine and feces), it is not known whether methyl eugenol was eliminated from the body, stored in distribution matrices such as adipose tissue, or a combination of the two. Animal studies suggest that methyl eugenol may be rapidly eliminated in urine as several metabolites. However, the presence of methyl eugenol in prefeed human samples, even after prolonged fasting, suggests that at least some methyl eugenol may be stored in distribution matrices that are at equilibrium with the blood.

Methyl eugenol was presented as a case study at the September 2004 International Biomonitoring Workshop (Albertini et al. 2006). Case study excerpts appear in a biomonitoring guidance document recently developed by European Centre for Ecotoxicology and Toxicology of Chemicals (2005). Here we present an overview of the current biomonitoring and other relevant data available on exposure to methyl eugenol and the use of these data in various environmental public health applications. Methyl eugenol was chosen as a case study for several reasons. Although human exposure potential is likely to be high, very limited human data and animal toxicity data are available. Furthermore, methyl eugenol is likely to be a common component of the diet. These existing data on methyl eugenol are confined to only a few target studies and a reasonably extensive NTP evaluation, albeit at comparatively high doses administered in bolus form. Animal toxicity studies indicate carcinogenicity at every level tested. Regardless, epidemiologic studies evaluating similar effects in humans have not been conducted, even though their evaluation would be comparatively easier than many other environmental chemicals. This case study illustrates and identifies some of the data gaps that need to be filled for the biomonitoring data on methyl eugenol to be integrated into a risk assessment.

## Pharmacokinetics

The human pharmacokinetics of methyl eugenol are not well defined. Schecter et al. (2004) reported that methyl eugenol has a serum half-life in humans of about 90 min. Little information is available on the human metabolism of methyl eugenol; however, there is some suggestion, based on data for estragole, a structural analogue, that it is similar to that in animals (Smith et al. 2002). In animals, methyl eugenol is quickly and completely absorbed, and the metabolism is regulated by dose. Methyl eugenol can undergo *O*-demethylation at low doses, and epoxidation or 1′-hydroxylation dominates at higher doses. A sulfated 1′-hydroxy intermediate that can undergo rearrangement to form an active carbonium species is believed to be the biologically active form of methyl eugenol. This was initially hypothesized as the species that caused an increase in unscheduled DNA synthesis (UDS; Chan and Caldwell 1992). Burkey et al. (2000) later confirmed this hypothesis by showing that methyl eugenol–induced UDS was obviated when cells were treated with pen-tachlorophenol, an inhibitor that prevented formation of the sulfated 1′-hydroxy species. Ultimately, several oxidative metabolites are excreted in urine, and there is some indication of conversion to CO_2_ (Smith et al. 2002). There is no indication of significant accumulation of methyl eugenol in any tissue.

## Toxicity Data

The available toxicology data indicate that methyl eugenol has relatively low acute toxicity by the oral route, with an acute LD_50_ (median lethal dose) in rats of about 1 g/kg (Beroza et al.1975) and acute percutaneous toxicity (LD_50_) of about 2 g/kg in rabbits (Beroza et al. 1975). There were body weight and hematologic effects in male and female F344 rats after gavage administration of 300 and 1,000 mg methyl eugenol/kg body weight (NTP 2000) for 14 weeks. The effects of methyl eugenol were also evaluated in male and female B6C3F_1_ mice after gavage administration of doses ranging from 10 to 1,000 mg methyl eugenol/kg body weight for 14 weeks (NTP 2000). There was significant mortality in the 1,000 mg/kg groups. The organs affected in the lower-dose groups included liver, glandular stomach, and nose (NTP 2000). Based on the results of the 14-week studies, male and female F344 rats and B6C3F_1_ mice received oral doses of 0, 37, 75, or 150 mg/kg/day for 5 days/week for 2 years. In addition, groups of male and female rats received 300 mg/kg/day methyl eugenol for 12 months, followed by a 12-month recovery period. It should be noted that none of the male rats in the 300-mg/kg/day group survived to the end of the 2-year study, and 16 of 50 female rats in this group survived to the end of the study.

The results of the rodent bioassay indicate that gavage administration of methyl eugenol resulted primarily in hepatocellular adenomas and carcinomas in male and female rats and mice (NTP 2000). There was some increase in the incidence of tumors in the glandular stomach in male and female rats and male mice (NTP 2000). In addition, there were increases in renal tubule adenoma, malignant mesothelioma, mammary gland fibroadenoma, and subcutaneous fibroma and fibrosarcoma in male rats (NTP 2000). Importantly, significant mortality was reported in the rats given doses of 150 mg/kg, with the entire male group dying and half of the female group dying before the end of the study (NTP 2000). The survival of vehicle control male and female rats was 40%, which approximates the historical control mortality for carboxymethyl cellulose vehicle. Based on the high mortality rate for the top-dose male and female rat treatment groups, it is plausible that the maximum tolerated dose was exceeded. Similarly, there was significant mortality among female mice treated with 150 mg/kg, with only two animals surviving to the end of the study compared to 31 female mice in the vehicle control group (NTP 2000). This suggests that high-dose female mice also received treatment that exceeded the maximum tolerated dose.

The NTP conducted a relatively extensive series of toxicokinetic studies with methyl eugenol in rats and mice (NTP 2000). The results of single-dose gavage administration studies indicate that maximum plasma concentrations were dose dependent and similar for rats and mice. However, the plasma half-life was shorter in mice than in rats (NTP 2000). The results of repeated-dose gavage studies in rats and mice indicate that methyl eugenol is rapidly absorbed, with most being eliminated in the urine, and some suggestion that it is eliminated more rapidly in mice than in rats (NTP 2000). Studies with [^14^C]-labeled methyl eugenol indicate that the primary organ for distribution in rats after either oral or intravenous administration is the liver (NTP 2000). The distribution in mice was different, with significant distribution to fat along with several organs, including liver, spleen, stomach, and ovaries (NTP 2000). In contrast to the data in rats, the methyl eugenol distribution in mice was similar to or greater in fat, spleen, ovary, and stomach than in liver.

The available genetic toxicology data suggest that methyl eugenol does not have significant genotoxic potential. With or without S9 metabolic activation, methyl eugenol was not mutagenic in *Salmonella typhimurium* strains TA98, TA100, TA1535, or TA1537 (NTP 2000). Methyl eugenol, with or without S9 metabolic activation, was not clastogenic when evaluated in cultured Chinese hamster ovary cells (NTP 2000), and it was not clastogenic in the bone marrow of male or female B6C3F_1_ mice (NTP 2000). One report indicates that methyl eugenol is cytotoxic to cultured primary hepatocytes from male F344 rats and female B6C3F_1_ mice (Burkey et al. 2000). In addition methyl eugenol caused a modest increase in UDS in primary rat and mouse hepatocytes (Burkey et al. 2000).

Both *in vitro* and *in vivo* DNA binding studies were conducted (Gardner et al. 1997; NTP 2000). Methyl eugenol, with or without S9 metabolic activation, was incubated with calf thymus DNA. The results of the study indicate that methyl eugenol itself had no detectable binding to DNA. When S9 from Arochlor 1254–treated mice or rats was used, DNA binding was higher than when non-induced S9 was used. The NTP reported that human S9 induced less DNA binding than either rat or mouse S9. Additional studies indicate that methyl eugenol forms DNA adducts *in vivo* (NTP 2000) and induces UDS *in vivo* (Burkey et al. 2000; Chan and Caldwell 1992; Gardner et al. 1997; Howes et al. 1990). The results of these studies support the hypothesis that the formation of a carbonium ion species that can react with DNA is the critical metabolite for methyl eugenol exposure. It is important to note that Smith et al. (2002) reported that none of the *in vivo* DNA adduct studies has been conducted at doses comparable to those found in the diet; in fact, the lowest dose used was about 10 mg/kg/day, which is comparable to the doses used in the NTP bioassay.

## Biomarker/Analytical Methods

Although several studies have evaluated bio-markers of methyl eugenol in animals, primarily rats, only one method has been reported to measure methyl eugenol in humans (Barr et al. 2000). The method used 4 g serum (the equivalent of a 10-mL whole-blood draw) to measure intact methyl eugenol. Isotopically labeled methyl eugenol was used as an internal standard. The methyl eugenol was isolated from the serum using a general solid-phase extraction and was measured in the extract using gas chromatography/high-resolution mass spectrometry. Quantification was achieved using isotope dilution calibration. Although the method was highly selective and sensitive, a residual contamination in the laboratory air and water made the analysis difficult. The method had appropriate sensitivity to measure methyl eugenol in serum, although the levels were quite low. An added benefit of measuring methyl eugenol in blood was the specificity of the marker for determining exposure to methyl eugenol itself. The measurement of metabolites can often be less selective because common metabolites may be derived from structurally similar chemicals. Urinary 1′-hydroxy methyl eugenol and its glucuronide-bound analogue are also considered potential biomarkers for human exposure to methyl eugenol, although no published methods exist for its measurement (Smith et al. 2002). Table 1 summarizes the validation parameters to be considered when evaluating biomarkers for methyl eugenol.

## Exposure Assessment

Limited information exists on specific sources of exposure to methyl eugenol. Because it is a component of many herbs and spices, differences in dietary habits along with the natural variation in levels of methyl eugenol in plants (Di Cesare et al. 2003; Smith et al. 2002) makes a definitive prediction of exposure difficult. Based on known levels in foods, spices, and herbs, the Flavor Extract Manufacturers Association (FEMA) estimated that methyl eugenol consumption from food is about 5–6 μg/kg/day (Smith et al. 2002). The FEMA group assumed that the top 10% of people consuming methyl eugenol consumed all of the methyl eugenol in commerce (Smith et al. 2002). In addition, use of essential oils as flavoring agents has resulted in a total methyl eugenol exposure of no more than about 10 μg/kg/day. However, ethnic or cultural dietary habits may result in substantially higher exposures to methyl eugenol; one estimate indicates as much as 250 μg methyl eugenol/kg/meal from a pesto meal (Miele et al. 2001).

The integrated exposure to methyl eugenol derived from the biomonitoring data indicates that serum levels have been measured as high as 390 pg/g and may be higher in some individuals depending on diet, genetics, body weight, and so forth (Barr et al. 2000; Schecter et al. 2004). The highest blood levels after consumption of about 216 μg of methyl eugenol in contained in gingersnap cookies was about 100 pg/g or 3.16 μg/kg (Schecter et al. 2004). These data and the highest level reported in the biomonitoring study (Barr et al. 2000) translate to exposures ranging from about 4–12 μg/kg/day, which is very similar to the estimates presented by Smith et al. (2002). Although exposure estimates based on the biomonitoring data are similar to those derived from food consumption, it should be noted that the biomonitoring data were derived from a subset of the general population and that population sampling did not account for dietary or other lifestyle factors that could influence methyl eugenol serum levels. This is supported by the fact that the highest serum levels in fasting individuals who consumed cookies containing methyl eugenol was only about one-fourth that of the highest serum levels reported in the NHANES population (Barr et al. 2000; Schecter et al. 2004). The fact that methyl eugenol has a relatively short half-life and that data regarding when food was consumed relative to when blood samples were collected from the NHANES subjects make it difficult to determine whether a steady-state was reached. This clearly highlights the need for both dietary information and temporal information when samples are collected. Furthermore, collection of multiple samples over relatively short periods of time would provide important additional data to help in the development of a human pharmacokinetic model. Development of a human pharmaco-kinetic model would be valuable for comparing the rodent toxicology data to human exposure. This could be further augmented with sample collection over longer intervals that, when coupled with the NHANES demographic information, would provide perspective on ethnic, gender, and/or racial exposure trends.

## Biomonitoring/Risk Assessment

Although no link between methyl eugenol consumption and health effects in humans has been identified, in part because of a limited number of studies in humans and an absence of epidemiology data, it is worthwhile to compare human exposure based on biomonitoring data to the available rodent bioassay data. A recent study provided some information regarding the pharmacokinetics of methyl eugenol in humans (Schecter et al. 2004). Volunteers consumed gingersnap cookies containing approximately 18 μg methyl eugenol per cookie, resulting in a total consumption of about 216 μg of methyl eugenol. The level of methyl eugenol in the blood peaked at about 15 min, and the half-life was estimated to be about 2 hr (Schecter et al. 2004). The highest exposure estimated from the Barr et al. (2000) study is as much as 4-fold greater than the highest exposure in the ginger-snap study (Schecter et al. 2004).

In contrast to the relatively low-dose exposures in humans, the lowest dose used in the 2-year rodent studies was 37,000 μg/kg/day. Thus, human dietary exposures are substantially lower than the lowest dose used in the NTP study. Even the high-consumption single-meal dose of about 250 μg methyl eugenol/kg (Miele et al. 2001) is almost 150-fold lower than the lowest dose used in the NTP study. It is important to note that the methyl eugenol biomonitoring data offer a starting point for examining the risk assessment paradigm as it applies to cancer risk assessment. Although the NTP rodent bioassay study design was intended primarily as a hazard identification tool, the results of such studies are often used to develop quantitative potency estimates for risk assessment. A number of different extrapolation methods have been developed and are used by state agencies such as the Office of Environmental Health Hazard Assessment in California, national agencies such as the U.S. Environmental Protection Agency, and international agencies such as the European Union and Health Canada for regulatory decision making. Typically for cancer risk assessment, linear extrapolation methods are used to derive a quantitative cancer risk or potency estimate, resulting in very low numerical values. Based on dietary consumption alone, it is likely that methyl eugenol exposure exceeds quantitative cancer potency estimates derived by linear extrapolation methods by several orders of magnitude. The exposure estimates outlined by Schecter et al. (2004) indicate that rodents had an increased liver cancer risk in the range of 17–50%. The authors indicated that human exposures would be about 10,000-fold lower than the exposures in the rodent bioassay. Although the projected dietary exposure is lower than the doses used in the rodent bio-assay, direct comparison is complicated because the animals received gavage doses as opposed to dietary administration, and there are no biomonitoring data in rodents after dietary consumption of methyl eugenol.

## Conclusions and Recommendations

Hazard identification data indicate that methyl eugenol induces hepatocellular carcinomas along with tumors at several other sites in rats and hepatocellular carcinoma in mice. However, the relevance of these results remains in question. The animals received substantial bolus doses that are not representative of typical methyl eugenol exposure. Relatively high doses of methyl eugenol that overwhelm the most common metabolic pathways have been suggested as the mechanism resulting in the formation of a reactive carbonium ion from the 1′-hydroxymethyl eugenol metabolite (Gardner et al. 1997; Rietjens et. al. 2005; Smith et al. 2002). This raises the possibility that a 1′-hydroxyl reactive metabolite is not formed at significant levels at lower exposures. It is important to note that given the lack of clinical or epidemiology data, there is no way to determine an association between human exposure to methyl eugenol and disease. Conducting human epidemiology studies to evaluate the association of diets containing a high level of methyl eugenol and any type of health outcome would provide valuable perspective. This clearly highlights the need for additional human data and the need to modify toxicology studies to include biomonitoring end points. The availability of biomonitoring data in humans and rodents after dietary consumption/low-dose exposure would greatly facilitate the integration of biomonitoring data into the risk assessment.

The potential for the formation of a reactive metabolite at high exposures as noted above suggests that more than one biomarker could be measured. The methyl eugenol biomonitoring data are based on detection of the parent compound in serum (Barr et al. 2000). Reports in the literature indicate a threshold for methyl eugenol tumor induction in rats (Waddell 2002). In addition Waddell et al. (2004) report that DNA adduct formation correlates with tumor induction in rats. The existing data support the conclusion that lower doses of methyl eugenol and other allyl alkoxy-benzenes undergo *O*-demethylation as the primary route of detoxification. This is supported by the report that microsomes from the livers of rats administered methyl eugenol at doses of 30–300 mg mg/kg autoinduced 1′-hydroxylation of methyl eugenol, whereas those of rats administered 10 mg/kg did not (Gardner et al. 1997). With relatively low (dietary) doses, it is likely that very little of the 1′-hydroxyl metabolite is formed; however, as doses increase, a shift in metabolism to this pathway occurs (Smith et al. 2002). Formation of the 1′-hydroxyl metabolite is particularly important because it is thought to form a carbonium ion that results in a reactive intermediate molecule (Burkey et al. 2000; Smith et al. 2002). Because the possibility of different metabolic pathways has been raised, additional perspective could be gained from biomonitoring the formation of the 1′-hydroxy and other metabolites at different doses, including comparisons of lower and higher doses of methyl eugenol. This type of information would help determine whether there is induction of a metabolic shift and would offer additional support for a threshold for methyl eugenol–induced effects, if a significant quantity of the reactive intermediate is formed only at higher doses.

The toxicokinetics of methyl eugenol after gavage have been reasonably well studied in animals, and a physiologically based pharmaco-kinetic model exists (NTP 2000). These data indicate that methyl eugenol is rapidly absorbed, metabolized, and excreted after bolus administration. There is one report that cytochrome P450 (CYP) E1 is responsible for 1′-hydroxylation in the rat (Gardner et al. 1997). For humans, there is some good preliminary information on the absorption and elimination of methyl eugenol (Schecter et al. 2004). The results of this study indicate that humans also rapidly absorb and eliminate methyl eugenol. However, only one dose was administered to a relatively small number of subjects. Obtaining additional data to develop a human physiologically based pharmaco-kinetic model would provide a tool for comparison and aid the interpretation of the rodent toxicity data. Recently, it has been reported that human CYP1A2 is responsible for 1′-hydroxylation at dietary concentrations and that CYP2C9, CYP2C19, and CYP2D6 participate at higher concentrations (Jeurissen et al. 2006). This further supports that there are distinct differences in the formation of the reactive metabolite at lower methyl eugenol exposures. In addition it suggests that there may be fundamental differences in the metabolism of methyl eugenol between rodents and humans. Because a single cytochrome P450 in humans appears to be primarily responsible for metabolism at low doses of methyl eugenol, it also raises the question of population heterogeneity. This highlights the need for obtaining additional metabolism and pharmacokinetic data in humans so that appropriate comparisons can be made to the existing animal data.

Finally, another important consideration is that the rodent bioassay was conducted using oral gavage as a means of administering methyl eugenol. There is limited relevance of oral gavage dosing for a material that is consumed at much lower levels in the diet. This has the net effect of presenting the animal with a large bolus dose of material that, as discussed above, may overwhelm detoxification pathways. The primary source of human exposure to methyl eugenol is most likely the diet. This would represent relatively low-level exposure that is likely to be well within both animal and human metabolic capacity. Evaluating the impact of route by administering methyl eugenol in the diet of rats and mice would add important information to our understanding of chronic dietary exposure to methyl eugenol. Tissue collection for biomonitoring information and determination of DNA adducts in the treated animals are included in the protocol. There should also be some consideration of gathering pharmaco-kinetic data after dietary administration because the existing data were gathered after gavage or intravenous administration.

This evaluation of methyl eugenol illustrates some of the questions that should be asked when attempting to integrate biomonitoring into risk assessment. It also highlights some of the data gaps that need to be filled to develop a risk assessment of methyl eugenol. Although there are a number of toxicology studies in animals, questions remain unanswered regarding the relevance of oral gavage administration of high-bolus doses used in the NTP studies, relative to the much lower doses received after dietary consumption of foods containing methyl eugenol. Recently there is some suggestion of a difference in metabolism of methyl eugenol between humans and rodents, and that significant amounts of the hypothesized critical metabolite occur at relatively high concentrations. Limited toxico-kinetic data in humans exist, and there may be differences in the primary detoxification pathways; additional toxicokinetic data are needed to gain a better understanding. This is particularly important because the 1′-hydroxy pathway is responsible for formation of the reactive carbonium ion. Furthermore, although there are very limited human health and no epidemiology data, no adverse human health effects have been associated with dietary consumption of methyl eugenol.

## Figures and Tables

**Figure 1:**
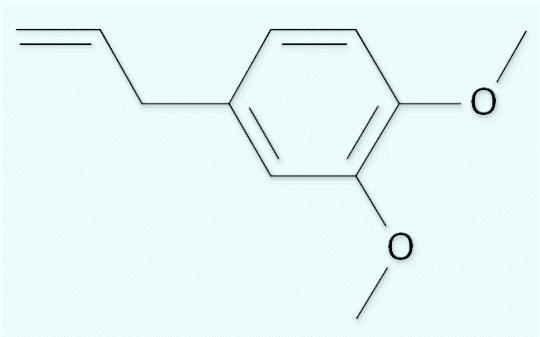
Methyl eugenol chemical structure.

**Table 1:** Evaluation of biomarkers for methyl eugenol.

Validation parameter	ME	1-OH-ME
Specificity of marker for exposure	Most specific	Most specific
Matrix for measurement	Blood	Blood
Alternative exposures that may result in presence of biomarker in matrix	None	None
Specificity of marker for predicting health outcome	Nonspecific	Nonspecific
Stability of marker in matrix	Very stable	Very stable
Data from multiple laboratories	No	No
Interlaboratory comparison	No	No

Abbreviations: ME, methyl eugenol; 1′-OH-ME, 1′-hydroxy methyl eugenol.
